# Early caregiver burden in owners of pets with suspected cancer: Owner psychosocial outcomes, communication behavior, and treatment factors

**DOI:** 10.1111/jvim.15905

**Published:** 2020-09-24

**Authors:** Marejka H. Shaevitz, Jeri A. Tullius, Robert T. Callahan, Christopher M. Fulkerson, Mary Beth Spitznagel

**Affiliations:** ^1^ Department of Veterinary Clinical Sciences Purdue University College of Veterinary Medicine West Lafayette Indiana USA; ^2^ Purdue University Center for Cancer Research West Lafayette Indiana USA; ^3^ Department of Psychology Kent State University Kent Ohio USA

**Keywords:** burden transfer, client factors, neoplasia, quality of life

## Abstract

**Background:**

Owners of companion animals with serious illnesses are likely to experience “caregiver burden.” This topic has not been fully evaluated in veterinary oncology.

**Objectives:**

To examine owners of a dog or cat with suspected cancer for relationships between *early* caregiver burden and (a) psychosocial factors: depression, stress, and quality of life; (b) owner communication behavior; and (c) specific pet treatment plan factors.

**Animals:**

None.

**Methods:**

This cross‐sectional, observational study recruited 164 owners of a cat or dog presenting for evaluation by a veterinary oncology service at a single referral institution. Measures of caregiver burden, psychosocial function, treatment plan elements, and demographics were collected online via owner self‐report. Medical records were reviewed to identify factors including diagnosis, medications, treatment schedules, and owner communications.

**Results:**

Caregiver burden correlated with higher stress (*r*_s_ = 0.40, *P* < .001), greater symptoms of depression (*r*_s_ = 0.50, *P* < .001), and lower quality of life (*r*_s_ = 0.39, *P* < .001). Pet treatment plan factors related to caregiver burden included changes in care routines, perception that compliance with new routines was challenging, and difficulty adhering to medication routines. There was low correlation between caregiver burden and owner‐driven communications (*r*_s_ = 0.15, *P* = .07).

**Conclusions and Clinical Importance:**

Findings suggest caregiver burden is similar in owners of pets with cancer and owners of pets with other diseases. Caregiver burden is present in the earliest stages of disease. Major correlates of burden including life‐disruptive treatments and schedules provide key areas for potential intervention by veterinary teams.

**Abbreviations**
CES‐D: Center for Epidemiology Studies Depression Scale
IRB: institutional review board
POAS: pet owner adherence scale
PSS: perceived stress scale
QLESQSF: quality of life enjoyment and satisfaction questionnaire‐short form
STROBE: Strengthening the Reporting of Observational Studies in Epidemiology
ZBI: Zarit Burden Interview

## 1 INTRODUCTION

Recent work indicates that companion animal owners experience depressive symptoms after a diagnosis of cancer in the animal.^1^ While depressive symptoms might be related to anticipated bereavement, so‐called “anticipatory grief,”^1^, ^2^ they might also be due in part to “caregiver burden.”^3^ Caregiver burden is a term traditionally used in human medicine to describe an individual's response to the challenges presented by caring for a sick human family member, and is related to poorer psychological and physiologic outcomes for the caregiver.^4^, ^5^, ^6^, ^7^ Caregiver burden has been recently documented in individuals caring for sick companion animals, particularly animals with chronic or terminal illnesses.^8^, ^9^ In these studies, burden was related to poor psychosocial functioning, including clinically meaningful levels of depressive symptoms and reduced quality of life.^8^, ^9^

There is a link between caregiver burden and specific elements of the companion animal's treatment plan, including several factors that the veterinary team can influence. For example, owners who report that their companion animal's disease management requires a major change to their daily routine or who feel like it is hard to adjust to these new routines report higher burden.^9^ Moreover, owners who experience higher burden are more likely to demonstrate certain behaviors in the context of interacting with their veterinary care team, including more frequent communications,^9^ conflict‐involving interactions, or both.^10^ These behaviors are in turn important contributors to veterinarian stress and burnout.^10^

Whether or not a companion animal develops cancer and whether that cancer is life‐threatening are things that cannot be changed. However, how an owner understands the situation, and what impact the diagnosis has on that individual's day‐to‐day life are things that the veterinary team can have significant influence over. Knowing how specific treatment factors affect caregiver burden and psychosocial well‐being could influence how the veterinary team approaches treatment planning and allow clinicians to employ strategies to reduce burden.

The purpose of our study was to examine owners of companion animals with suspected cancer evaluated by an oncology service at a referral hospital, as these owners represent a unique demographic relative to owners previously studied,^8^, ^9^ for the relationship between burden and (a) psychosocial factors of depressive symptoms, stress, and quality of life, (b) clinic communication behavior, and (c) specific factors of treatment plan complexity including subjective experience (eg, difficulty adjusting to new routines) and objective report (eg, number and frequency of medications). We hypothesized that higher burden would be associated with poorer psychosocial functioning, greater owner‐driven communication with the clinic, and changes or challenges in the treatment plan.

## 2 MATERIALS AND METHODS

This research was conducted and reported in accordance with Strengthening the Reporting of Observational Studies in Epidemiology (STROBE) criteria.^11^ The study protocol was reviewed and approved by the institutional review boards (IRBs) of Purdue University and Kent State University. All participants provided informed consent for study participation.

### 2.1 Sample

Participants were owners of newly registered dogs and cats evaluated by the oncology service at a large academic specialty hospital. Inclusion criteria to enroll in the study were that the participant must be ≥18 years of age, able to comprehend and respond to questions in English, and currently living with and providing care for a dog or cat presenting to the center for diagnosis and possible treatment. Exclusion criteria included failure to complete the protocol (ie, beginning the protocol but discontinuing before the end). Participants were not excluded if they completed the protocol but declined to answer specific questions as this was an option required by the IRB.

### 2.2 Demographic information

Participant age (continuous, with drop‐down response format), sex (multiple choice response format: Male, Female), education level (multiple choice response format: 8 years or less, 9‐11 years, High school graduate, Associate's degree [or equivalent], Bachelor's degree [or equivalent], Master's degree, Doctoral degree), ethnicity (multiple choice response format: African‐American, Asian American, Caucasian, Latin American, Native American, Other), percentage of caregiving responsibility (continuous, slide bar format), annual income (multiple choice response format: under $25 000 per year, $25 000‐$49 999 per year, $50 000‐$74 999 per year, $74 999‐$100 000 per year, over $100 000 per year), number of people in the household (drop down menus with choices from 0 to 20), number of companion animals in the household (drop down menus with choices from 0 to 20), companion animal species (multiple choice response format: Dog, Cat, Other), and companion animal duration of disease (multiple choice response format: <1 month, 1‐6 months, 6‐12 months, 12+ months) were collected from all participants by self‐report.

### 2.3 Record review

Data extraction from veterinary records was conducted by the research team (Purdue University and Kent State University). Data from the time of the initial visit (when questionnaires were completed) were retrieved for: (a) diagnosis, (b) number of daily medications prescribed (number), (c) times per day the pet receives medication (number), (d) whether the pet was prescribed a new medication for presenting diagnosis before referral (yes/no), (e) whether the pet was prescribed a new medication that required a schedule change, or had a schedule change to an existing medication before referral, for presenting diagnosis (yes/no), (f) estimated treatment cost (number), and (g) miles from caregiver's home address to treatment center (number). In addition, number of incoming telephone contacts (no inbound email, text, or other communication method to the clinic was available to clients at the time) from the owner to the service in the 30 days after study enrollment were counted. The 30‐day period after the initial meeting was selected for the purposes of retention, as several of the presenting conditions were terminal.

### 2.4 Study measures and instruments

A brief description of measures is presented below; the purpose and psychometric properties of these instruments have been described in greater detail.^8^, ^9^ All scoring of questionnaires was conducted in aggregate and in accordance with measure instructions.

#### 2.4.1 Caregiver burden

The Zarit Burden Interview (ZBI)^3^ adapted for use with companion animals^8^ includes 18 items rated on a scale of 0 (never) to 4 (nearly always). Questions reflect subjective (eg, feelings of guilt, anger, frustration) and objective (eg, physical health, social life, financial impact) burdens of caregiving. Example subjective questions include “Do you feel angry when you are around your pet?” and “Do you feel embarrassed over your pet's behavior?”^8^ Example objective questions include “Do you feel your health has suffered because of your involvement with your pet?” and “Do you feel that you don't have enough money to care for your relative in addition to the rest of your expenses?”^8^ Higher scores indicate greater level of burden. A cutoff of 18 on the ZBI adapted for use with companion animals indicates clinically meaningful caregiver burden.^8^ Cronbach's *α* for the current sample was *α* = .89.

#### 2.4.2 Perceived stress

The Perceived Stress Scale^12^ is a 10‐item scale addressing degree of current stress on scale of 0 (never) to 4 (very often) with reversal of item scores as indicated. Items ask about frequency of feelings of being nervous, stressed, and difficulty controlling irritation. There is not a standard cutoff to indicate increased perceived stress. Scores typically range from 12 to 14 (range of possible scores, 0‐40), with higher scores indicating higher levels of stress.^9^ Cronbach's *α* for the current sample was *α* = .91.

#### 2.4.3 Depressive symptoms

The Center for Epidemiology Studies Depression scale^13^ assessed presence of depressive symptoms. This 20‐item instrument asks about various symptoms and presentations of depression, which the respondent rates from 0 (rarely or none of the time) to 3 (all of the time), with reversal of item scores as indicated. A higher CES‐D score indicates greater presence of depressive symptoms, and a score of ≥16 indicates risk for clinical depression.^9^ Cronbach's *α* for the current sample was *α* = .91.

#### 2.4.4 Quality of life

The Quality of Life Enjoyment and Satisfaction Questionnaire‐Short Form (QLESQSF)^14^, ^15^ is a 16‐item measure of quality of life which asks the respondent to indicate level of satisfaction across multiple domains (eg, mood, work, social life) on a scale from 1 (very poor) to 5 (very good). Thirteen (13) of the 16 questions from this measure were used, as previously described.^9^ Two items that are not included in total scoring were omitted, and a third item was removed due to sensitive content not considered integral to the research question. No cutoff exists for this measure; however, higher score indicates better quality of life, and the measure has evidence of acceptable psychometric properties.^9^ Cronbach's *α* for the current sample was *α* = .95.

#### 2.4.5 Treatment factors

Selected items from the pet owner adherence scale (POAS)^16^ were utilized. The POAS is a measure of owner perceptions of a companion animal's disease and treatment. Although designed to examine adherence, this scale addresses several issues relevant to caregiving for a companion animal, including treatment needs and owners' subjective perceptions of the treatment. Seven (7) items related to how well the owner feels the companion animal's disease, medications, and treatment routines have been explained, and how difficult it is to comply with treatment regimens were utilized. Responses are scored on a scale of 1 (strongly disagree) to 5 (strongly agree). No cutoff exists for this measure; however, higher scores indicate greater perceived difficulty related to the treatment plan. Cronbach's *α* for the selected items used in the current sample was *α* = .71.

### 2.5 Recruitment and data collection procedures

Data were collected between July 2018 and March 2019 for this cross‐sectional, observational study. All participants were owners registering a new companion animal (cat or dog) for evaluation and potential treatment with the above‐described oncology service. Owners were asked by clinical staff about their interest in research participation during the clinical evaluation. Interested individuals then discussed enrollment in the study with a research assistant. Owners who agreed to participate were asked to provide informed consent to complete study questionnaires and allow research personnel to extract information from the pet's records. They were provided an electronic device equipped with a Qualtrics‐based instrument including the above‐described measures, and were asked to complete measures during “down time” at their companion animal's appointment. Any owners willing to participate but unable to complete measures during this period were sent an email link to the protocol to complete it remotely online. Participation was solely on a volunteer basis, and was not reimbursed.

### 2.6 Power analysis

Prior work examining caregiver burden in owners of companion animals^8^, ^9^, ^10^ demonstrated that the adapted ZBI correlates with measures of depression, stress, quality of life, difficulty of the treatment plan, and nonbillable owner contacts between *r* = 0.31 to 0.59. Using a significance level of *α* = .05 and power *π* = 0.8 for a medium effect (*r* = 0.30), a conservative sample size of 85 participants was needed. Over‐sampling was planned due to the expectation that the current sample would consist largely of owners presenting their companion animals early in the disease process, which could be associated with lower overall levels of caregiver burden.^17^

### 2.7 Statistical analysis

Demographic variables (frequencies or mean ± SD as appropriate) were first analyzed. Primary study variables were evaluated for normality using histograms. The ZBI and data extracted via chart review demonstrated skewed distributions, kurtotic distributions, or both and POAS variables are ordinal in nature; as such, nonparametric tests were applied for all analyses. Spearman rank‐order correlations were conducted to examine caregiver burden (adapted ZBI) and psychosocial function variables of depressive symptoms (CES‐D), perceived stress (PSS), and quality of life (QLESQSF). Next, in the owners with animals that had a confirmed diagnosis of cancer, frequency of owner‐driven communications over the 30 days after completion of measures was examined using Spearman's rho correlation with the adapted ZBI. Spearman's rho was also used to examine relationships between adapted ZBI and variables related to the owner's understanding of disease and difficulty enacting the treatment plan (selected POAS variables), as well as either point‐biserial correlations (binary variables from chart review: presence or absence of prior medication, prescription of new medication for the presenting diagnosis, and whether a new medication schedule was required) or Spearman's rho (number of daily medications, times per day the pet receives medication, estimated treatment cost, and distance from owner's home to treatment center in miles). The family‐wise *α* level for all significance tests was set at .05, with the sequentially rejective Holm‐Bonferroni correction applied to minimize type I error.^18^ All statistical analyses were conducted using SPSS Version 26.0 (IBM Corp, Armonk, New York).

## 3 RESULTS

### 3.1 Sample

Of 310 owners approached between June 22, 2018 and March 6, 2019, a total of 173 owners (55.8%) enrolled in the study; after exclusion for failure to complete the online protocol, the analytic sample was 164. Participants were generally middle‐aged (median 48.55 years), female (67.7%), Caucasian (95.1%), and had a college degree or higher (76.8%). The majority of companion animals were dogs (92.1%) with the remainder being cats (7.9%). See Table 1 for sample demographic make‐up. For analyses involving owner‐driven communications, a further exclusion for no diagnosis of cancer (n = 11) was applied for a total of 153 in this subsample, as these individuals would have no reason to continue with the oncology service.

**TABLE 1:** Participant demographics

Demographic variable	Full sample (N = 164)
*Owner*	
Age (mean ± SD)	48.6 ± 13.9
Sex (percent of sample)	
Female	111 (67.7%)
Male	52 (31.7%)
Declined to respond	1 (0.6%)
Race/ethnicity (percent of sample)	
White/Caucasian	156 (95.1%)
Latin American/Hispanic	4 (2.4%)
Asian American	1 (0.6%)
Declined to respond	3 (1.8%)
Education (percent of sample)	
High school or lower	38 (23.2%)
College degree	77 (47.0%)
Graduate degree	47 (28.7%)
Declined to respond	2 (1.2%)
Percent of caregiving responsibility (mean ± SD)	81.7 ± 21.3
*Household*	
Annual household income (percent of sample)	
<$25 000	5 (3.0%)
$25 000‐$49 999	22 (13.4%)
$50 000‐$74 999	39 (23.8%)
$75 000‐$100 000	41 (25.0%)
>$100 000	54 (32.9%)
Declined to respond	3 (1.8%)
People in household (percent of sample)	
1	28 (17.1%)
2	73 (44.5%)
3	28 (17.1%)
4	23 (14.0%)
5	7 (4.3%)
6	2 (1.2%)
Declined to respond	3 (1.8%)
Animals in household (percent of sample)	
1	43 (26.2%)
2	42 (25.6%)
3	35 (21.3%)
4	24 (14.6%)
5	9 (5.5%)
6	4 (2.4%)
7	1 (0.6%)
8	3 (1.8%)
Declined to respond	3 (1.8%)
*Companion animal*	
Species (percent of sample)	
Dog	151 (92.1%)
Cat	13 (7.9%)
Age (mean ± SD)	8.95 ± 3.15
Sex (percent of sample)	
Male	8 (4.9%)
Male neutered	71 (43.3%)
Female	2 (1.2%)
Female spayed	81 (49.4)
Declined to respond	2 (1.2%)
Diagnosis (percent of sample)	
Lymphoma	25 (15.2%)
Urothelial carcinoma	21 (12.8%)
Soft tissue sarcoma	15 (9.1%)
Mast cell tumor	12 (7.3%)
AGASACA	7 (4.3%)
Histiocytic sarcoma	6 (3.7%)
Adenocarcinoma (nonanal sac)	4 (2.4%)
Other carcinoma	4 (2.4%)
Hemangiosarcoma	4 (2.4%)
Melanoma	4 (2.4%)
Squamous cell carcinoma	3 (1.8%)
Leukemia	2 (1.2%)
Multiple cancer types	9 (5.5%)
Other neoplasm	9 (5.5%)
Undiagnosed tumor	28 (17.1%)
Noncancer	11 (6.7%)
Benign tumor	3 (1.8%)
Noncancer diagnosis	8 (4.9%)
Duration of disease (percent of sample)	
<1 month	100 (60.9%)
>1 month	39 (23.7%)
Declined to respond	25 (15.2%)

Abbreviation: AGASACA, apocrine gland anal sac adenocarcinoma.

### 3.2 Caregiver burden and psychosocial function

Descriptive statistics for each questionnaire are shown in Table 2. Spearman rank‐order correlation analyses in the full sample revealed significant relationships between adapted ZBI scores and scores for each of the psychosocial function questionnaires (*P* < .001 for all; Table 2). Greater caregiver burden was associated with greater symptoms of depression (*r*_s_ = 0.50), higher levels of perceived stress (*r*_s_ = 0.40), and lower quality of life (*r*_s_ = 0.39).

**TABLE 2:** Descriptive statistics and Spearman correlation matrix for caregiver burden (adapted ZBI) and questionnaires used to assess psychosocial factors of depressive symptoms (CES‐D), stress (PSS), and quality of life (QLESQSF)

Measure	Median/Interquartile range/Min‐max	ZBI	CES‐D	PSS
ZBI (adapted)	**11/10.75/1‐34**	—	—	—
CES‐D	**9/13/0‐53**	0.50	—	—
PSS	**13/10/0‐36**	0.40	0.71	—
QLESQSF	**52.5/16.75/13‐65**	−0.39	−0.68	−0.69

*Note*: Values are shown as *r*_s_. *P* ≤ .001 for all correlations and remain significant after Holm‐Bonferroni correction. The bolded numbers are descriptive statistics.

Abbreviations: CES‐D, Center for Epidemiological Studies Depression Scale; PSS, perceived stress scale; QLESQSF, quality of life enjoyment and satisfaction questionnaire, short form; ZBI, Zarit Burden Interview.

### 3.3 Caregiver burden and frequency of owner‐driven communications

In the subset of owners whose companion animal was diagnosed with cancer (n = 153), Spearman correlation analysis between the number of owner‐driven communications in the 30 days after measure completion and adapted ZBI scores revealed a small‐to‐moderate association (*r*_s_ = 0.15, *P* = .07).^19^

### 3.4 Caregiver burden and chart review: Treatment, cost, and treatment center distance

Adapted ZBI scores and treatment variables extracted from the medical chart are presented in Table 3; correlations of the greatest magnitude were presence of medication required at the time of consult (*r*_s_ = 0.14, *P* = .08) and number of prior medications required (*r*_s_ = 0.13, *P* = .09).^19^ See Table 3.

**TABLE 3:** Results of Spearman rank‐order and point‐biserial correlation analyses between caregiver burden (adapted ZBI) and ratings for individual treatment variables assessed via POAS and chart review

Chart review	Adapted ZBI
1. Medication required before presenting diagnosis	0.14^†^
2. New medication prescribed for presenting diagnosis	−0.03
3. New medication schedule required for presenting diagnosis	−0.02
4. Number of daily medications required for presenting diagnosis	0.13^†^
5. Number of times per day medications administered	0.09
6. Treatment cost estimate	−0.04
7. Distance from owner's home to treatment center	−0.10
**POAS items**	
1. My daily routine has changed because of because of my pet's illness/disease	0.44**
2. It is challenging to follow new rules/routines needed for management of my pet's illness/disease	0.46**
3. My pet's illness/disease has been explained to me in detail	0.03
4. My pet's pharmacological treatment (medication) has been explained to me in detail	−0.24*
5. It is simple to follow my pet's medication routine	−0.38**
6. My pet's medications appear to be effective	−0.23*
7. The rules/routines for managing my pet's illness/disease have been explained to me in detail	−0.15

*Note*: Values are shown as *r*_s_ except point‐biserial *r* for Chart Review items 1 to 3.

Abbreviations: POAS, Pet Owner Adherence Scale (copyright © 2015 Zita Talamonti et al..); ZBI, Zarit Burden Interview.

^*^ *P* < .05.

^**^ *P* ≤ .001.

^†^ *P* < .1. Significant *P* values remain significant after Holm‐Bonferroni correction.

### 3.5 Caregiver burden and owner understanding of disease, perception of treatment plan

Results of Spearman correlation analyses between adapted ZBI scores and responses to the selected POAS item demonstrated several relationships, with greatest magnitude of correlation observed for ratings related to the routines involved in managing the animal's illness, including change in daily routine (*r*_s_ = 0.44, *P* ≤ .001), perception that new routines are challenging to follow (*r*_s_ = 0.46, *P* ≤ .001), and difficulty following the medication routine (*r*_s_ = 0.38, *P* ≤ .001). Additional relationships were seen between ZBI scores and owner understanding of disease management, including perception that medications are ineffective (*r*_s_ = 0.23, *P* < .05), and perception that the pet's pharmacological interventions have not been explained in detail (*r*_s_ = 0.24, *P* < .05). See Table 3.

## 4 DISCUSSION

While previous work has included owners of companion animals with cancer in a general hospital setting,^9^ the goal of our study was to examine owners of companion animals with suspected cancer evaluated in a referral hospital setting for the relationship between caregiver burden and owner psychosocial function, communication behavior, and treatment factors. Our study builds upon and extends previous work on burden by investigating burden in a referral hospital setting, representing a demographically unique group relative to samples previously studied, and including objective review of medical records to determine the relationship between burden and treatment‐related factors. Burden was present in the current sample, and significantly related to higher levels of stress, greater symptoms of depression, and lower quality of life, and also showed a small‐to‐moderate, meaningful correlation with the number of owner‐driven communications to the clinic. As hypothesized, several treatment plan factors were related to caregiver burden.

Results suggest that the burden of caregiving for a companion animal with cancer or suspected cancer can be distressing for the owner. These findings are consistent with previous studies, which showed that caregiver burden correlates positively with measures of depressive symptoms and stress, and negatively with measures of quality of life.^8^, ^9^ Similarly, the small to moderate relationship^19^ observed between caregiver burden and greater owner‐driven communications to the clinic parallels findings from past work,^9^ demonstrating a connection between the owner's caregiver burden and their communication behavior with the veterinary clinic. The magnitude of correlations in the current work suggests these relationships are meaningful,^19^ but the relationship strengths detected here are generally smaller than was found in past work using a general veterinary hospital sample.^8^, ^9^ The smaller effects in our study could reflect the different disease presentations in a general hospital vs oncological setting, but might also be due to timing. Data for our study were collected at an initial appointment evaluating for possible cancer, and more than half of our sample reported duration of disease lower than 1 month. In comparison, prior work reporting similar relationships between burden and psychosocial function^8^ was conducted in a sample in which the majority of owners reported duration of disease lasting a year or longer. Similarly, past work examining the relationship between caregiver burden and owner‐driven communications^9^ did so over the course of a full year, whereas our study only examined 30 days. Work in human samples shows that duration of disease predicts caregiver burden,^20^, ^21^ and duration of caregiving is correlated with burden in companion animal caregiving samples.^17^ Average burden in the current sample was lower than has been observed in prior samples,^8^, ^9^, ^17^ suggesting possible restricted range of burden within this sample, which would in turn impact the strength of these relationships. Importantly, directionality of these findings cannot be assumed, that is, we do not know that caregiver burden is driving owners to feel more depressed or stressed, or to make more frequent phone calls—it is possible that those with higher levels of psychosocial dysfunction and those who would have been more likely to make frequent calls are simply experiencing caregiving as more burdensome. Work from human caregiving suggests the former relationship, that is, that caregiver burden is the instigating factor,^22^ but longitudinal research is needed to definitively determine directionality.

The likely impact of caregiver burden on both the owner (ie, psychosocial distress) and veterinary provider (ie, additional owner communications) underscores the importance of considering how the owner's caregiver burden might be reduced. To this end, we examined variables associated with the treatment plan. Results indicated burden was most significantly correlated with perceptions of challenge in managing new medication routines and rules, and impact of medication routines on the owner's daily schedule. In addition, the sense that medications had been explained and were effective also negatively associated with burden. Additionally, a correlation was observed between burden and whether medications were required before the diagnosis and the number of medications given for the current illness; while relatively small in magnitude, these relationships might ultimately bear important consequences.^19^ These findings extend prior work,^9^ in which a small sample from a general hospital clientele showed significant correlations in the relationship between caregiver burden and change in routine as a result of a companion animal's illness and perception that the routine was difficult. Our study has a larger sample relative to the previous study, which contributed greater power to detect significant relationships. Our study also extends prior work by adding the objective element of record review and more specific detail (eg, number of medications). Again, the cross‐sectional nature of this work precludes definitive comments on directionality; however, an intuitive interpretation of these relationships is that owners feel greater distress when challenged to juggle new schedules and routines to provide care for their companion animal, or frustrated, guilty, or both when there are many medications to administer, particularly if the animal is difficult to medicate and efforts to do so are not uniformly successful. Work from human medicine shows that a shift in perceived role can contribute to caregiver burden^23^; if the owner experiences the treatment plan as altering the nature of their relationship with their companion animal from primarily companion to primarily caregiver or nurse, this might also have an effect to increase burden.

Our study carries important implications for owners and for practitioners. It is essential to understand that owners caring for a companion animal with cancer or suspected cancer, even in early stages, might show caregiver burden. This burden might have consequences for the owner in terms of poorer psychosocial function, and might also impact veterinary providers through greater demand for nonbillable attention and support (ie, burden transfer).^10^ Recognizing, understanding, and reacting appropriately could help reduce burden for the owner and minimize potential for burden transfer to veterinary personnel. This might be accomplished by recognizing key treatment plan elements that are related to burden, and finding ways to minimize potential impact on the owner. Based on our study, the following treatment‐related recommendations might be helpful in reducing caregiver burden and burden transfer.

1. Collaborate with owners to determine how to fit medication or other treatment schedules into their natural daily routines. Developing clear and concrete treatment plans alongside the owner that honors existing daily demands and abilities might help reduce caregiver strain.^24^ Moreover, owners might be more likely to adhere to reasonably accomplishable plans, which could result in improved pet outcomes as well. This recommendation is relevant for all fields of veterinary medicine, but might have particular relevance for specialty medicine and oncology in particular given the intensity of therapeutic regimens and limited access to treatment facilities that can provide appropriate care.
2. Minimize the number of medications a pet receives, even if on the same schedule. Our study extends past research by showing that caregiver burden shows a small relationship with the quantity of medications administered to a companion animal. Veterinarians can impact this by (a) selecting medication sizes to employ the fewest number of individual tablets or capsules to achieve medically effective drug doses; (b) resisting prescription of multiple medications for the same indication when fewer can accomplish the same goal; (c) discontinuing redundant or unnecessary medications; or (d) communicating with owners about tapering or discontinuation schedules, and providing a concrete plan the owner can follow with clear expectations about the treatment timeline. While this recommendation is relevant in all contexts, it might be particularly relevant for oncology teams who frequently offer demanding treatment protocols, but also where protocols' demands might diminish over time such as by moving from weekly to every‐other‐week treatments.
3. Clearly explain both the indications and expected results from medications to minimize owner perceptions of inefficacy or futility when overt effects might not be expected, and provide resources to help owners achieve better understanding of treatment protocols. Our study revealed a link between perceiving an intervention as ineffective and increased burden. Although veterinarians should optimally work with owners to develop the most effective treatment regimen possible, there might be times when the clinician and owner define “effectiveness” differently. For instance, for certain cancers and treatment protocols, lack of progression or stable disease is a positive outcome that might not be recognized by owners whose traditional conception of effectiveness might be cure or tumor reduction.^25^ Providing clear information regarding expectations and outcomes might reduce ambiguity and frustration surrounding interventions with effects that are less recognizable to a layperson.

Working with owners around their experiences of caregiving might help both owners and veterinary teams. Identifying alternative support systems such as caregiver groups or access to social workers might be useful in reducing caregiver burden (and in turn, burden transfer), though research is needed to determine if this is the case. The link between burden and frequent client initiated contacts suggests that implementation of a team approach including veterinary social workers and client liaisons might be beneficial for clients and in reducing the strain placed on veterinarians and technicians.^24^ Moreover, acknowledging and validating the existence of burden and associated emotions provides an opportunity for the veterinary team to offer validation and understanding, and enhance collaborative caregiving with owners.^26^

Limitations of the current work are acknowledged. A primary limitation is in the cross‐sectional examination of owners in the earliest stages of their companion animal's illness. As noted above, because duration of care is positively related to burden, the current sample might not have had sufficient time to develop significant burden. Issues such as having a new treatment plan, covering costs for expensive diagnostics, expensive treatment, or both, or driving a far distance to the oncology center might not have been significantly related to burden at this time point simply because they had not yet emerged as known stressors for these owners. Burden is likely to develop with time,^20^, ^21^ perhaps after the owner has received bills or repeatedly undertakes a long drive to the treatment center. Longitudinal work is needed to examine the primary contributors to development of burden in a veterinary oncology setting over time. Another limitation might be found in that participants were not consecutively enrolled clients of the oncology service, but rather, recruited on a voluntary basis during their initial visit to the oncology center. Not all individuals elected to participate in the study. The sample might include individuals who were particularly motivated to describe their experiences, which could reduce generalizability. Furthermore, because data were collected at a single referral hospital, they might not be fully representative of the population of owners of pets with cancer, as our population was comprised of mostly female (although a greater percent of male participants was included relative to past work^8^), mostly white, mostly well‐educated, and entirely English‐competent individuals. Our participants were also biased strongly toward dog‐ownership over cat‐ownership, reflecting the population served by the referral institution, but not necessarily reflecting the population of companion animals with cancer or suspected cancer.

In summary, the current work demonstrates the existence of caregiver burden as early as the initial referral visit for caregivers of pets with cancer or suspected cancer. It also confirms relationships between burden and owner psychosocial variables, including stress, depression, and reduced quality of life, as well as potential impact on veterinary medical professionals via greater need for communication. Major correlates of burden including life‐disruptive treatments and schedules provide key areas for potential intervention by veterinary medical teams.

## CONFLICT OF INTEREST DECLARATION

Authors declare no conflict of interest.

## OFF‐LABEL ANTIMICROBIAL DECLARATION

Authors declare no off‐label use of antimicrobials.

## INSTITUTIONAL ANIMAL CARE AND USE COMMITTEE (IACUC) OR OTHER APPROVAL DECLARATION

Approved by Purdue University Institutional Review Board, Protocol #1802020199, without full review as category 2 exempt research. The Kent State University Institutional Review Board reviewed and acknowledged that this protocol was approved without full review as category 2 exempt research.

## HUMAN ETHICS APPROVAL DECLARATION

Approved by Purdue University Institutional Review Board, Protocol #1802020199.
